# Regular Meeting of the Fulton County Medical Society, May 4, 1916

**Published:** 1916-05

**Authors:** 


### REGULAR MEETING OF THE FULTON COUNTY MEDICAL SOCIETY, MAY 4, 1916.

Dr. M. T. Davis read a paper on: "Some Observations of the Heart." This appears in this issue of the JOURNAL.

In discussing the paper Dr. W. W. Young called attention to the newer methods of testing the functional capacity of the heart. Functional tests of the kidneys and liver have been developed very greatly within the last few years and Dr. Young thinks that functional tests on the heart will yield equally as gratifying results as the other functional tests. In other words, we should be able to determine exactly how much the heart is able to standing in the way of exercise and instruct the patient accordingly. By doing this we will accomplish a great deal towards lessening the suffering and lengthening the lives of those patients with chronic heart disease.

Dr. T. C. Davison exhibited a case which he had done a partial gastrectomy for a tumor in the pyloric region of the stomach. The interesting feature of this case as stated by Dr. Davison is that the patient had a typical attack of pellagra sometime before the operation and immediately following the operation the patient had another attack of the same trouble. However the patient recovered from the acute attack of pellagra and from the operation and is now in apparently perfect health. The patient gained much in weight and is now able to eat and digest her food properly, whereas before the operation her digestion was very much impaired.

Dr. J. S. Derr exhibited X-Ray plates taken of this case before the operation in which he was able to demonstrate the presence of the tumor in the stomach. Dr. Derr stated that from the X-Ray plates he was satisfied that the tumor was not malignant.

Dr. Allen H. Bunce described the tumor removed by Dr. Davison stating that it involved the entire pyloric region of the stomach. Upon pathological examination he found the tumor to be non-malignant. The tumor was composed of connective tissue and was probably developed upon the site of an old ulcer.

Dr. L. C. Fischer desired to know whether or not the tumor might have been of syphilitic origin and asked if a Wassermann Test had been done on the patient. Dr. Fischer stated that the cases of gumma of the stomach which he reported a year ago had remained well after taking anti-syphilitic treatment.

In reply to Dr. Fischer's question, Dr. Bunce stated that a Wassermann Test done on the blood of the patient was negative.

Dr. Geo. M. Niles read a paper on the "Diagnosis of Duodenal Ulcer X-Ray and Otherwise."

Dr. Niles also exhibited a number of X-Ray plates showing the characteristic X-Ray findings in duodenal ulcer and also showed how this condition could be distinguished from other pathological conditions of the stomach and duodenum.

Dr. Derr discussed the X-Ray findings in this case and stressed the importance of the X-Ray diagnosis of various gastrointestinal conditions.

Dr. O. O. Fanning asked if the cases reported by Dr. Niles had been confirmed by operation.

Dr. L. C. Fischer stated that in his work he had found the X-Ray findings to be of very great aid in making a correct diagnosis. Dr. Fischer stated that the X-Ray, laboratory and clinical findings should be considered together in each case so as to be able to make a more correct diagnosis before undertaking operative procedure and thanked Dr. Niles for his interesting report of the diagnostic value of the X-Ray.

In closing Dr. Niles stated in reply to the question of Dr. Fanning that the patients reported by him had been operated on and the findings at that time were identical with the X-Ray diagnosis.

Dr. Allen H. Bunce read a paper: "Observations on Vaccine Theraphy—A summary of the views of some of the leading men of the U. S."

Dr. Arch Elkin stated that the indiscriminate use of vaccine was a thing which would bring vaccine therapy into disrepute. During his recent service at the Grady Hospital Dr. Elkin observed carefully a series of cases of typhoid fever which were treated with typhoid vaccine. He stated that from these he was almost convinced that vaccines were of positive value in the treatment since those cases receiving vaccines were of shorter duration and had less complications than those not receiving vaccines. He thinks that the usual failure to get results from tuberculins is because the majority of tuberculins which are on the market are not potent. He states that he has himself taken 100 times the amount of the average dose of tuberculin from one of the reliable manufacturers without any appreciable effect.

Dr. E. C. Thrash stated that he thinks the chief difficulty in getting results with vaccines is due to the fact that some other organism is grown instead of the one causing the disease. In other words, it is his opinion that very frequently vaccines are made from bacteria which happen to be present in the infection but are not the real cause of the disease process. He states that if the real offending organism is obtained and a proper vaccine made from it that results will be obtained in practically all cases. He advises the more frequent use of blood cultures since by this method there is less opportunity of getting non-pathogenic organisms. He thinks the best results are obtained by giving the vaccines intravenously.

Dr. O. L. Miller asked if any replies had been obtained with reference to vaccines in the prevention and treatment of influenza.

Dr. W. N. Adkins asked if any replies had been received with reference to the use of pertussis vaccine.

In closing Dr. Bunce stated that no replies had been received which mentioned either influenza or pertussis vaccine.

